# Reduced *USP22* Expression Impairs Mitotic Removal of H2B Monoubiquitination, Alters Chromatin Compaction and Induces Chromosome Instability That May Promote Oncogenesis

**DOI:** 10.3390/cancers13051043

**Published:** 2021-03-02

**Authors:** Lucile M. Jeusset, Brent J. Guppy, Zelda Lichtensztejn, Darin McDonald, Kirk J. McManus

**Affiliations:** 1 Research Institute in Oncology & Hematology, CancerCare Manitoba, Winnipeg, MB R3E0V9, Canada; jeussetl@myumanitoba.ca (L.M.J.); brentguppy@me.com (B.J.G.); Zelda.Lichtensztejn@umanitoba.ca (Z.L.); 2 Department of Biochemistry & Medical Genetics, University of Manitoba, Winnipeg, MB R3E0J9, Canada; 3 Department of Oncology, University of Alberta, Edmonton, AB T6G2H7, Canada; mhendzel@ualberta.ca

**Keywords:** chromosome instability, *USP22*, H2B ubiquitination, H2Bub1, quantitative imaging microscopy, cancer, BUB1, chromatin compaction, STED, mitosis

## Abstract

**Simple Summary:**

Chromosome instability (CIN) promotes cancer initiation and progression, but its genetic origins remain largely unknown. As aberrant mitotic processes such as chromatin compaction defects can induce CIN, characterizing the molecular determinants of mitotic fidelity may reveal novel CIN genes. In vitro, monoubiquitination of histone H2B at lysine 120 (H2Bub1) impairs chromatin compaction, while in vivo H2Bub1 is rapidly depleted in early mitosis. USP22 is a major deubiquitinating enzyme catalyzing H2Bub1 removal in interphase and may also be responsible for H2Bub1 depletion in mitosis. To assess whether mitotic removal of H2Bub1 is required for mitotic fidelity, we employ complementary genetic and single-cell imaging microscopy approaches to assess the impact reduced *USP22* expression has on H2Bub1 abundance, chromatin compaction and chromosome stability. We show that *USP22* deficiency impairs H2Bub1 removal and induces chromatin compaction defects. Further, we identify *USP22* as a novel CIN gene, indicating that *USP22* deletions in tumors may drive CIN and contribute to oncogenesis.

**Abstract:**

Chromosome instability (CIN) is an enabling feature of oncogenesis associated with poor patient outcomes, whose genetic determinants remain largely unknown. As mitotic chromatin compaction defects can compromise the accuracy of chromosome segregation into daughter cells and drive CIN, characterizing the molecular mechanisms ensuring accurate chromatin compaction may identify novel CIN genes. In vitro, histone H2B monoubiquitination at lysine 120 (H2Bub1) impairs chromatin compaction, while in vivo H2Bub1 is rapidly depleted from chromatin upon entry into mitosis, suggesting that H2Bub1 removal may be a pre-requisite for mitotic fidelity. The deubiquitinating enzyme USP22 catalyzes H2Bub1 removal in interphase and may also be required for H2Bub1 removal in early mitosis to maintain chromosome stability. In this study, we demonstrate that siRNA-mediated USP22 depletion increases H2Bub1 levels in early mitosis and induces CIN phenotypes associated with mitotic chromatin compaction defects revealed by super-resolution microscopy. Moreover, *USP22*-knockout models exhibit continuously changing chromosome complements over time. These data identify mitotic removal of H2Bub1 as a critical determinant of chromatin compaction and faithful chromosome segregation. We further demonstrate that *USP22* is a CIN gene, indicating that *USP22* deletions, which are frequent in many tumor types, may drive genetic heterogeneity and contribute to cancer pathogenesis.

## 1. Introduction

Each year, cancer affects over 18 million new individuals and causes ~9.5 million deaths worldwide [1], highlighting the need for a greater understanding of the molecular mechanisms promoting initiation and progression of the disease. Chromosome instability (CIN) is an aberrant phenotype observed in virtually all cancer types, including up to 85% of colorectal cancer cases [2,3]. CIN is characterized by an increase in the rate at which whole chromosomes or large chromosome fragments are gained or lost [4,5]. Conceptually, CIN increases the rate at which key cancer-associated genes, such as oncogenes and tumor suppressor, apoptotic or DNA repair genes, are gained, lost or altered [3,6,7,8]. Thus, while high levels of CIN can compromise viability, intermediate levels are proposed to increase intra-tumor genetic heterogeneity and promote the emergence of more aggressive clones within a tumor [9,10,11,12]. Accordingly, it is not surprising that CIN is often associated with aggressive cancers, the acquisition of multi-drug resistance and poor patient prognosis [2,13,14,15,16,17,18]. Despite these associations, the genetic changes giving rise to CIN are largely unknown, and the CIN genes associated with intermediate levels of CIN remain to be identified [2,19,20,21].

CIN often arises from defects within the molecular mechanisms that govern mitotic fidelity [22], such as aberrant regulation of chromatin compaction [23]. The basic unit of chromatin is the nucleosome, composed of 146 base pairs of DNA coiled around a histone octamer constituted by two copies of each of the core histones, H2A, H2B, H3 and H4 [24,25]. In interphase, dynamic changes in the three-dimensional organization and compaction of the nucleosomes regulate chromatin accessibility to control DNA-associated processes, including transcription, replication and DNA damage repair [26,27]. Post-translational histone modifications, such as acetylation, methylation and ubiquitination, transiently regulate chromatin accessibility by altering histone–histone and histone–DNA interactions, or by recruiting effector proteins [28]. In mitosis, additional levels of chromatin compaction enable individualization of sister chromatids and impart chromatin with sufficient stiffness to withstand the pulling forces exerted by microtubules emanating from the centrosomes, in order to ensure the accurate partitioning of chromosomes into daughter cells [25,29]. Multiple histone modifications (reviewed in [23]), such as phosphorylation of histone H3 on serine residues 10 and 28, are temporally regulated during mitosis and are essential to achieve higher-order chromatin compaction and maintain mitotic fidelity, and the misregulation of these mitosis-specific dynamics induces CIN [23,30,31,32,33].

Histone H2B monoubiquitination on lysine 120 (H2Bub1) occurs on ~1% of interphase nucleosomes [34] and its presence or absence dynamically regulates gene expression [35,36] and DNA repair [37,38,39]. Recently, H2Bub1 was identified to exhibit mitosis-specific dynamics—maximal abundance occurs in interphase and is rapidly depleted during prophase, to remain undetectable from prometaphase until cytokinesis/early G1, when H2Bub1 abundance begins to increase [40]. Like many histone modifications, these mitosis-specific dynamics suggest that the removal of H2Bub1 in mitosis may be an essential prerequisite that ensures mitotic fidelity [23,30,31,32,33]. In support of this possibility, in vitro biophysical assays demonstrate that the presence of H2Bub1 disrupts higher-order chromatin compaction through electrostatic interactions between specific ubiquitin residues and the surface of the nucleosome histone octamer [41,42,43]. As higher-order chromatin compaction is essential for accurate chromosome segregation during mitosis [29,32,44], the timely removal of H2Bub1 may be critical to ensure accurate chromosome compaction and proper segregation. At least nine deubiquitinating enzymes may catalyze H2Bub1 removal in humans, and while these enzymes may be partially redundant, emerging evidence indicates that they predominantly function in distinct cellular processes (reviewed in [45]). Among these candidates, USP22 (Ubiquitin Specific Peptidase 22) is arguably the best characterized enzyme as its depletion corresponds with global increases in H2Bub1 abundance in multiple cellular contexts [46,47,48]. Thus, we posit that USP22 is the primary enzyme orchestrating the timely removal of H2Bub1 during mitosis, which is essential to enable appropriate mitotic chromosome compaction and preserve chromosome stability.

Using a series of complementary genetic and single-cell quantitative imaging (QuantIM) approaches, we examined the functional impact aberrant H2Bub1 removal during early mitotic stages has on chromatin compaction and chromosome stability. Using karyotypically stable cell lines, we first show that *USP22* silencing corresponds with increases in H2Bub1 abundance within prophase chromosomes that are accompanied by mitotic chromatin compaction defects as revealed by super-resolution microscopy. In addition, QuantIM revealed that *USP22* silencing compromises mitotic fidelity and induces CIN phenotypes, including increases in micronucleus formation, changes in nuclear areas and alterations in chromosome numbers. To determine the long-term impact aberrant H2Bub1 regulation has on CIN, two *USP22*-knockout (KO) clones were generated with CRISPR-Cas9. Clones were assessed over a 10-week period and each exhibited dynamic CIN phenotypes relative to controls. In agreement with USP22 depletion being a pathogenic event in cancer, we determined that *USP22* deletions occur frequently in many cancer types and shallow/deep deletions are associated with worse progression-free survival. Collectively, our findings demonstrate that H2Bub1 removal in early mitosis is critical for higher-order chromatin compaction, accurate chromosome segregation and the maintenance of chromosome stability, and they further identify *USP22* as a novel CIN gene, consistent with diminished *USP22* expression being a pathogenic event contributing to oncogenesis.

## 2. Results

### 2.1. USP22 Silencing Compromises H2Bub1 Removal in Early Mitosis and Alters Mitotic Chromatin Compaction

**Figure 1:**
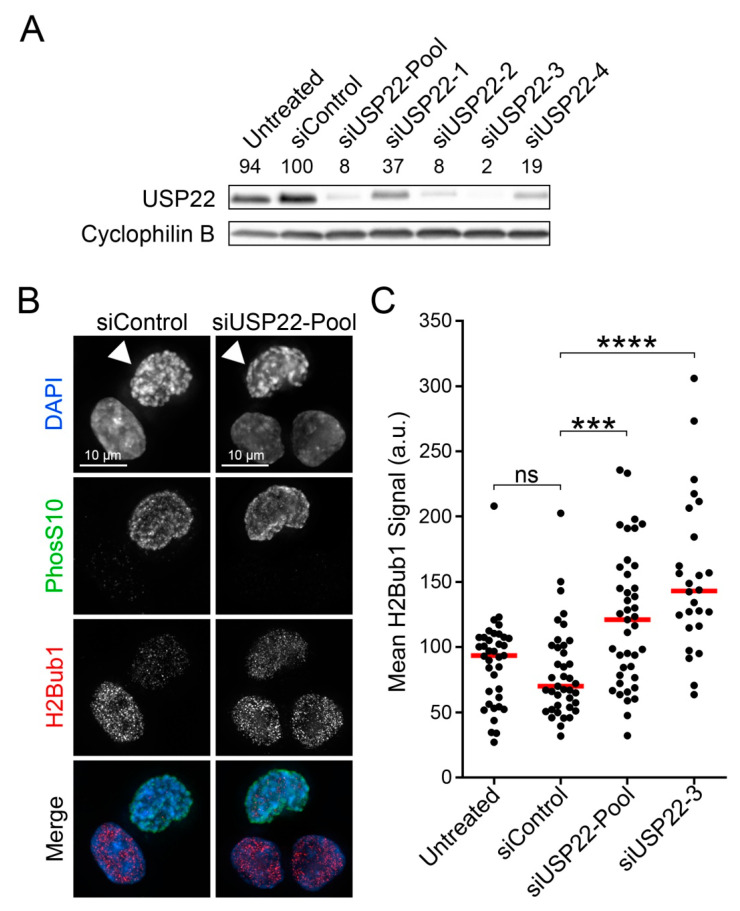
*USP22* silencing induces increases in global H2Bub1 levels in prophase cells. (**A**) Western blot presenting USP22 abundance following silencing in HCT116 with either individual (siUSP22-1, -2, -3 and -4) or pooled *USP22* (siUSP22-Pool) siRNAs and controls (untreated or siControl); cyclophilin B serves as the loading control. Semi-quantitative analyses were performed and the normalized USP22 levels are presented relative to siControl (100%). (**B**) Representative high-resolution 3D images (maximal intensity projection) of prophase HCT116 cells (arrowheads) immunofluorescently labeled for PhosS10 and H2Bub1 following silencing. For quantitative purposes, all images were acquired using identical exposure times. (**C**) Dot plot presenting the mean H2Bub1 signal intensity/cell, with red bars identifying median signal intensities. Mann–Whitney tests identify significant increases in mean H2Bub1 intensities (i.e., protein expression levels) following *USP22* silencing relative to siControl (N = 2; n > 25 cells/condition; ns *p*-value > 0.05; *** *p*-value < 0.001; **** *p*-value < 0.0001).

Under normal conditions, H2Bub1 levels are rapidly lost during prophase and prometaphase [40] and its presence impairs higher-order chromatin compaction in vitro [41,42,43]. In many organisms, proper mitotic chromosome compaction is essential for chromosome segregation and mitotic fidelity [23,31,32,33]. Collectively, these observations raise the possibility that the timely removal of H2Bub1 may be an essential pre-requisite for mitotic fidelity in human cells. To test this possibility, we sought to determine whether reduced *USP22* expression impairs H2Bub1 removal specifically within prophase chromosomes. HCT116 cells were carefully chosen for this work, as they are karyotypically stable and harbor wild-type (diploid) copies of the genes encoding the H2B ubiquitination (*RNF20* and *RNF40*) and deubiquitination (*USP22*) machinery [49]. To reduce *USP22* expression, four individual siRNAs (siUSP22-1, -2, -3 and -4) and an siRNA pool (siUSP22-Pool) were assessed by Western blot, with USP22 levels reproducibly reduced to <10% of the non-targeting control (siControl) with siUSP22-Pool, siUSP22-2 and siUSP22-3 (Figure 1A). Western blot analysis of H2Bub1 abundance within asynchronous *USP22*-silenced and control cells indicates that *USP22* silencing induces a moderate increase (1.1- to 1.5-fold) in the global abundance of H2Bub1 (Figure S1). To specifically assess H2Bub1 abundance within prophase nuclei, asynchronous cells were fixed, co-immunofluorescently labeled for H2Bub1 and a mitosis-specific marker (histone H3 Serine 10 phosphorylation [PhosS10]), counterstained (DAPI) and subjected to QuantIM (Figure 1B). As predicted, *USP22* silencing induced significant increases in median H2Bub1 signal intensities specifically within prophase cells (siUSP22-Pool, 1.7-fold; siUSP22-3, 2.0-fold) relative to siControl (Figure 1C; Table S1), identifying USP22 as a major deubiquitinating enzyme responsible for H2Bub1 removal during mitosis.

**Figure 2:**
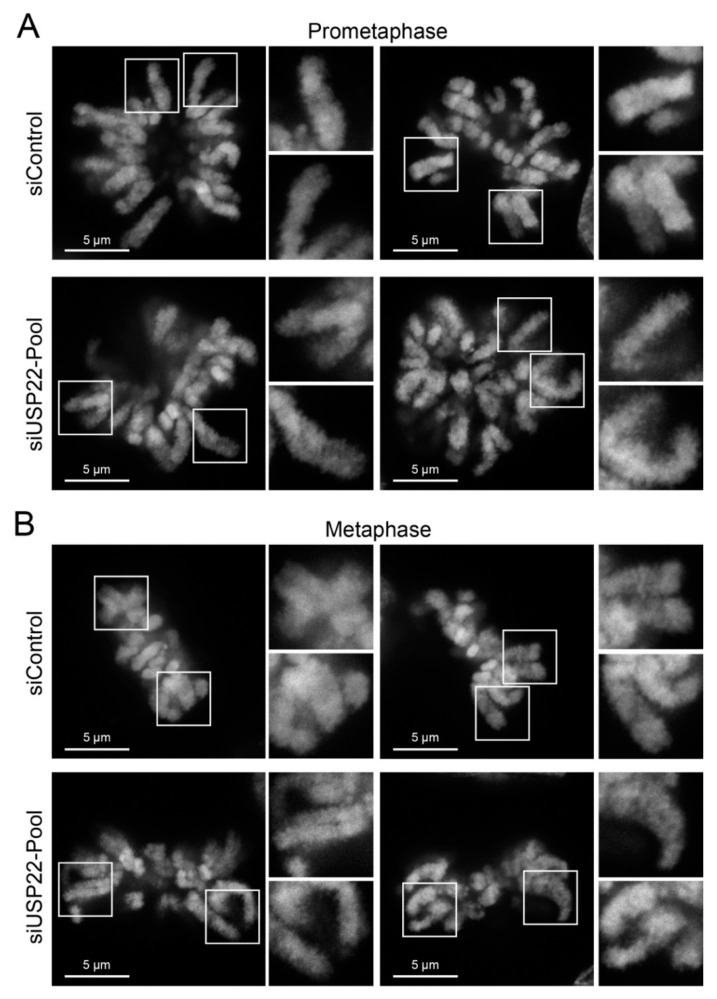
*USP22* silencing induces chromatin compaction defects in mitotic HCT116 cells. (**A**) Representative STED images of prometaphase chromosomes from siControl (top) and *USP22*-silenced conditions (bottom). White bounding boxes identify the magnified regions presented in the right-hand panels. Note the less dense and more granular staining patterns present within the *USP22*-silenced conditions, which are indicative of chromosome compaction defects. (**B**) STED images comparing chromosome compaction within siControl (top) and *USP22*-silenced (bottom) metaphase cells with similar differences in staining patterns (density and granularity) to those indicated above.

Next, super-resolution stimulated emission depletion (STED) microscopy (Figure 2, Table S2) was employed to determine whether the increases in H2Bub1 abundance accompanying *USP22* silencing induce mitotic chromatin compaction defects. Examination of the images revealed overt phenotypic differences between control and *USP22*-silenced conditions. All control chromatids in prometaphase (100%) and metaphase (100%) were densely stained with clearly defined borders, whereas 44% of prometaphase and 50% of metaphase *USP22*-silenced cells exhibited poorly defined chromatids, with diffuse edges and granular chromatin structures indicative of compaction defects [44]. Collectively, these findings show that reduced *USP22* expression corresponds with increases in H2Bub1 abundance and aberrant chromatin compaction (i.e., structural defects) specifically within mitotic chromatids, which supports the possibility that reduced *USP22* expression may adversely impact chromosome stability.

### 2.2. Reduced USP22 Expression Compromises Mitotic Fidelity

Over the past two decades, several groups have shown that aberrant mitotic chromosome compaction increases chromosome congression and/or segregation errors [29,32,44]. To determine whether the compaction defects observed above are sufficient to impact normal chromosome dynamics, *USP22*-silenced and control cells were assessed for congression and segregation errors in metaphase and anaphase cells (Figure S2), respectively. While no increases in congression errors or chromatin bridges were apparent, there was a 3.6-fold increase in lagging chromosomes within *USP22*-silenced cells (Table 1) that is consistent with reduced *USP22* expression and impaired H2Bub1 removal compromising chromosome segregation and mitotic fidelity.

**Figure 3:**
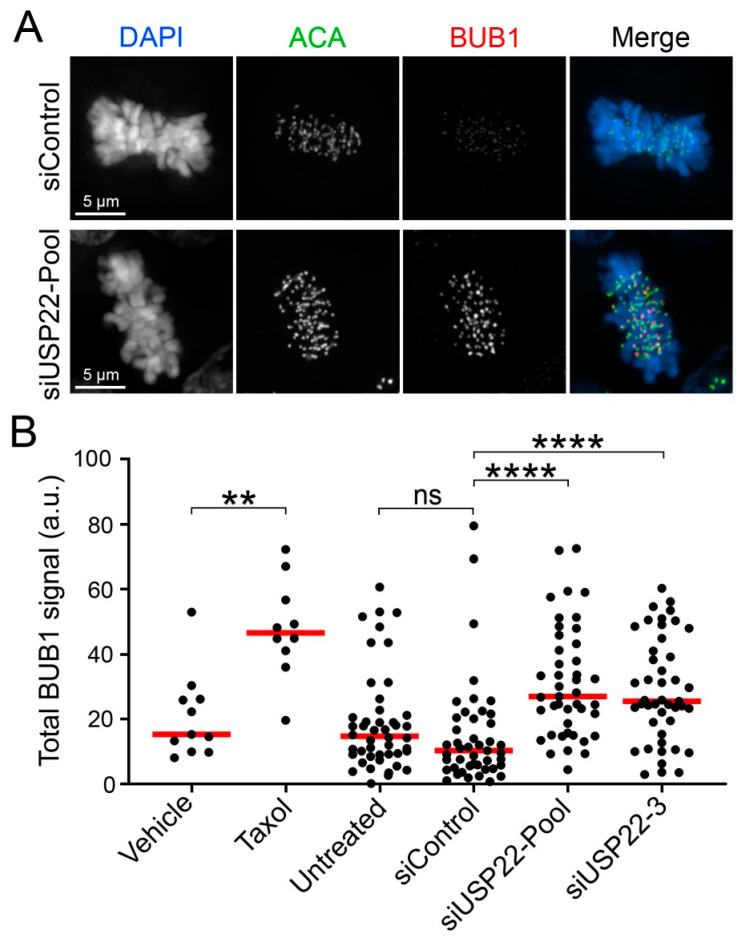
*USP22* silencing increases BUB1 recruitment to kinetochores in metaphase cells. (**A**) Representative deconvolved 3D images (maximal intensity projection) of metaphase HCT116 cells immunofluorescently labeled for ACA and BUB1 following *USP22* silencing. For quantitative purposes, all images were acquired using identical exposure times. Note the visual increase in BUB1 signal intensities within the *USP22*-silenced condition (bottom panel) relative to siControl condition (top panel). (**B**) Dot plot showing the total kinetochore-associated BUB1 signal intensity/cell from a minimum of 40 metaphase cells per silencing condition (minimum 10 cells for vehicle control and Taxol conditions), with red bars identifying median intensities. Mann–Whitney tests identify significant increases in total BUB1 intensities (i.e., levels) following Taxol treatment (positive control) relative to vehicle control and following *USP22* silencing relative to siControl (N = 2; n ≥ 10 for vehicle and Taxol controls; n > 40 for silencing conditions; ns *p*-value > 0.05; ** *p*-value < 0.01; **** *p*-value < 0.0001).

Chromatin compaction confers physical stiffness to mitotic chromosomes that counteracts pulling forces exerted by the mitotic spindle and produces inter-kinetochore tension between sister chromatids [50,51,52]. As inter-kinetochore tension exerts a critical role in the detection and correction of erroneous kinetochore–microtubule attachments [53], altered chromatin compaction may impair inter-kinetochore tension and the correction of aberrant kinetochore–microtubule attachments, resulting in chromosome segregation defects such as lagging chromosomes. To gain insight into the underlying mechanism accounting for the increase in lagging chromosomes within USP22-depleted cells, inter-kinetochore tension was assessed by quantifying BUB1 abundance specifically at kinetochores. BUB1 is a kinase normally recruited to tensionless kinetochores that can activate the spindle assembly checkpoint (SAC) [54,55,56]. Asynchronous cells were co-immunofluorescently labeled with anti-centromeric antibody (ACA) and BUB1, and QuantIM was used to assess the total signal intensities (i.e., abundance) of the kinetochore-associated BUB1 foci within metaphase cells (Figure 3A). Taxol served as a positive control since it inhibits microtubule dynamics, resulting in tensionless kinetochores and increased BUB1 recruitment [56]. As expected, Taxol treatment induced significant increases in BUB1 signal intensities, which were also observed following *USP22* silencing (pooled or individual siRNAs), but not with the siControl (Figure 3B and Figure S3; Table S3). These data indicate that the timely removal of H2Bub1 by USP22 during mitosis is essential for the establishment of inter-centromeric tension in metaphase.

As recruitment of BUB1 at the kinetochores can activate the SAC and delay anaphase entry [54,55], we sought to evaluate whether the increase in kinetochore-associated BUB1 observed in *USP22*-silenced cells is sufficient to delay anaphase onset. HCT116 cells expressing H2B-GFP were silenced and live cells were imaged every 2 min 30 s to monitor mitotic progression. The time from nuclear envelope breakdown to anaphase entry was similar within *USP22*-silenced cells (23.1 ± 5.2 min) and controls (21.7 ± 5.0 min; Figure S4), indicating that the defects in inter-centromeric tension revealed by increased BUB1 recruitment are not sufficient to induce a prolonged SAC activation; however, they may be sufficient to impair correction of erroneous kinetochore–microtubule attachments. In particular, merotelic attachments (i.e., simultaneous attachment of a kinetochore to both spindle poles) do not activate the SAC and generate lagging chromosomes specifically in anaphase when left uncorrected. As the mechanisms responsible for correction of merotelic attachments may be exquisitely sensitive to subtle tension defects in metaphase [53,57,58], impaired chromatin compaction and altered inter-centromeric tension within *USP22*-silenced cells may compromise these repair mechanisms, resulting in increased frequency of segregation defects.

### 2.3. USP22 Silencing Induces CIN-Associated Phenotypes

**Figure 4:**
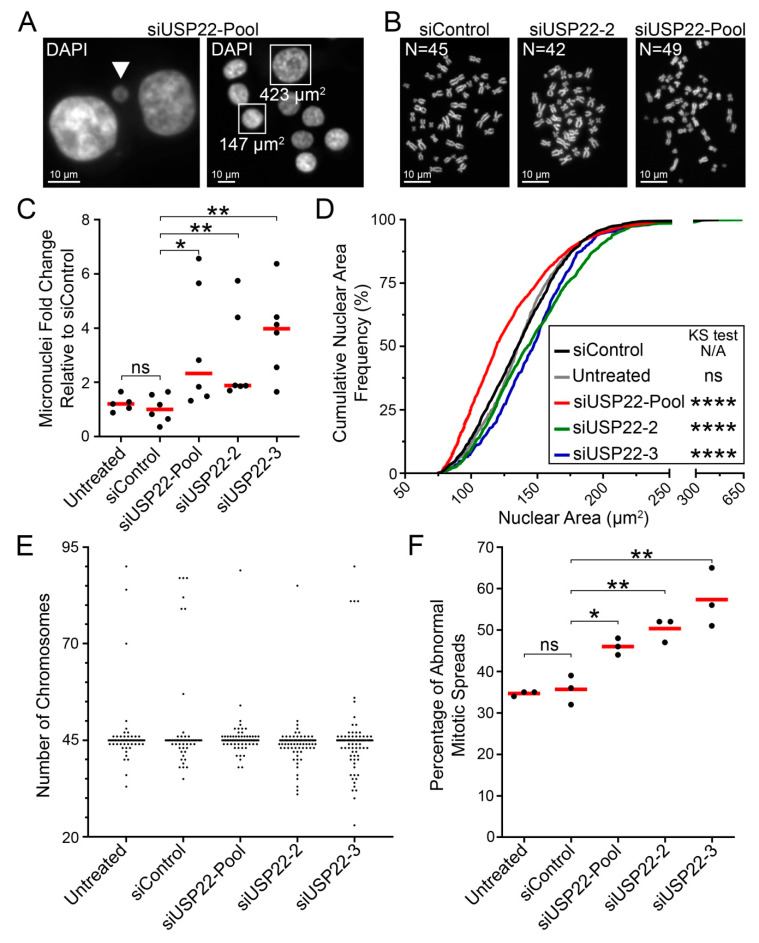
*USP22* silencing induces CIN phenotypes in HCT116. (**A**) Representative images of *USP22*-silenced nuclei displaying a micronucleus (left, arrowhead) and nuclear area heterogeneity (right). Bounding boxes identify two nuclei for which the respective areas are indicated. (**B**) Representative images of mitotic chromosome spreads exhibiting normal (left) chromosome numbers, losses (middle) and gains (right). Note that the modal chromosome number is 45 for HCT116. (**C**) Dot plot presenting the fold change in micronucleus formation relative to the median of siControl; red bars identify median values. Mann–Whitney tests identify statistically significant increases in micronucleus formation following *USP22* silencing relative to siControl (N = 2; n ≥ 5; minimum 100 nuclei analyzed/replicate; ns [not significant] *p*-value > 0.05; * *p*-value < 0.05; ** *p*-value < 0.01). (**D**) Two-sample KS tests reveal significant changes in cumulative nuclear area distribution frequencies following *USP22* silencing relative to siControl (N = 2; minimum 600 nuclei analyzed/condition; N/A not applicable; ns *p*-value > 0.01; **** *p*-value < 0.0001). (**E**) Representative dot plot presenting the number of chromosomes enumerated from 100 mitotic chromosome spreads/condition (N = 3). An increase in the frequency of chromosome losses (N < 45) and gains (N > 45) is observed following *USP22* silencing. (**F**) Dot plot showing the frequency of abnormal mitotic chromosome spreads (N ≠ 45) following *USP22* silencing relative to controls. Experiments were repeated three times, with the red bars identifying the mean. Student’s *t*-tests reveal significant increases in the mean number of mitotic chromosome spreads with aberrant chromosome numbers relative to siControl (N = 3; n = 100; ns *p*-value > 0.05; * *p*-value < 0.05; ** *p*-value < 0.01).

As chromosome mis-segregation is a major mechanism behind CIN [22], reduced *USP22* expression and altered H2Bub1 regulation during mitosis may represent novel determinants of CIN. To explore this possibility, *USP22*-silenced cells were subjected to QuantIM to assess CIN-associated phenotypes, including micronucleus formation and changes in nuclear areas (Figure 4A). Briefly, micronuclei are small, extra-nuclear bodies containing mis-segregated chromosomes or chromosome fragments and are hallmarks of CIN [5,59,60,61], while changes in nuclear areas typically correlate with changes in chromosome complements [62]. Thus, changes (i.e., increases in cell-to-cell heterogeneity) in nuclear areas and micronucleus formation are used to identify putative CIN genes [5,62,63] that can be subsequently validated with mitotic chromosome enumeration (Figure 4B). HCT116 cells were again selected, as they are karyotypically stable and have been employed extensively in similar CIN-based studies [20,40,62,64,65]. As predicted, *USP22* silencing induced significant increases (siUSP22-Pool, 2.3-fold; siUSP22-2, 1.9-fold; siUSP22-3, 4.0-fold) in median micronucleus formation relative to siControl (Figure 4C; Table S4). Similarly, reduced expression also induced changes in median nuclear areas for siUSP22-Pool (119.5 μm^2^), siUSP22-2 (143.0 μm^2^) and siUSP22-3 (148.0 μm^2^) compared to siControl (136.0 μm^2^) or untreated (136.0 μm^2^) cells (Figure 4D; Table S5). Furthermore, two-sample Kolmogorov–Smirnov (KS) tests revealed statistically significant increases (siUSP22-2, siUSP22-3) and a decrease (siUSP22-Pool) in cumulative nuclear area frequency distributions relative to siControl (Figure 4D; Table S5) that are consistent with reduced *USP22* expression inducing CIN. The divergent changes (increases vs. decreases) in nuclear areas are not unexpected, as they are in agreement with the heterogeneous nature of CIN, which includes both gains and losses in chromosome numbers. To explore this possibility, mitotic chromosome spreads were generated in HCT116 cells (modal chromosome number = 45) and manual chromosome enumeration revealed that *USP22* silencing corresponded with increases in chromosome losses and gains relative to siControl (Figure 4E). Further, the total frequency of abnormal mitotic spreads (N ≠ 45) increased significantly from 1.3- to 1.6-fold following silencing with siUSP22-Pool (46%), siUSP22-2 (50%) and siUSP22-3 (57%) relative to siControl (36%) and untreated (35%; Figure 4F; Table S6) conditions. Collectively, these findings establish that reduced *USP22* expression increases the frequency of chromosome losses and gains and thus identify *USP22* as a novel CIN gene in HCT116 cells.

### 2.4. USP22 is a Conserved CIN Gene

**Figure 5:**
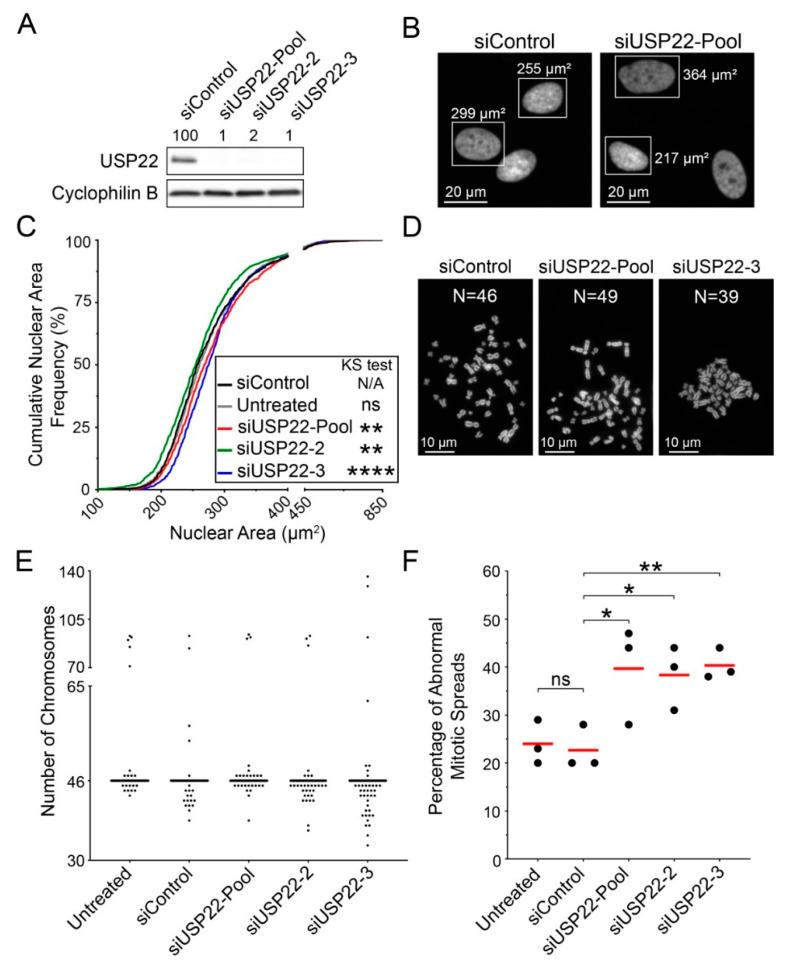
*USP22* silencing induces CIN phenotypes in hTERT cells. (**A**) Western blot showing decreases in USP22 abundance following silencing; cyclophilin B is the loading control. Semi-quantitative analyses were performed and the normalized USP22 levels are presented relative to siControl (100%). (**B**) Representative images of control (left) and *USP22*-silenced (right) nuclei. Bounding boxes identify nuclei for which the respective areas are indicated. Note an increase in nuclear area heterogeneity within the *USP22*-silenced cells. (**C**) Two-sample KS tests reveal statistically significant differences in cumulative nuclear area distribution frequencies following *USP22* silencing relative to siControl (N = 2; n > 900 nuclei analyzed/condition; ns *p*-value > 0.01; ** *p*-value < 0.01, **** *p*-value < 0.0001). (**D**) Representative images of mitotic chromosome spreads exhibiting normal (left) chromosome numbers, gains (middle) and losses (right). Note that the modal chromosome number is 46 for hTERT. (**E**) Dot plot presenting the number of chromosomes enumerated from a single representative experiment (N = 3). An increase in the frequency of chromosome losses (N < 46) and gains (N > 46) is observed following *USP22* silencing. (**F**) Dot plot showing the significant increases in the frequency of abnormal mitotic chromosome spreads (N ≠ 46) following *USP22* silencing relative to controls. The red bars identify the mean values of the three replicates. Student’s *t*-tests comparing means relative to siControl (N = 3; n = 100; N/A not applicable; ns *p*-value > 0.05; * *p*-value < 0.05; ** *p*-value < 0.01).

To assess the conserved nature of *USP22* as a CIN gene, similar experiments were performed in a second karyotypically stable cell line. Human telomerase-immortalized fibroblasts, hTERT, were purposefully selected as they are a non-malignant and non-epithelial cell line model (modal chromosome number = 46) that have been employed in similar CIN-based studies [62,65,66,67]. *USP22* silencing efficiencies were first established (Figure 5A). While QuantIM did not identify reproducible increases in micronucleus formation following silencing (Figure S5), significant differences in cumulative nuclear area distribution frequencies were observed, with shifts towards both smaller (siUSP22-2) and larger distributions (siUSP22-Pool and siUSP22-3) (Figure 5B,C; Table S5). Mitotic chromosome spreads also revealed that *USP22* silencing corresponded with increases in both chromosome losses (N < 46) and gains (N > 46, Figure 5D–E), with the average frequency of aberrant spreads increasing significantly from 1.7- to 1.8-fold following *USP22* silencing (siUSP22-Pool, 40%; siUSP22-2, 38%; siUSP22-3, 40%) relative to siControl (23%) (Figure 5F; Table S6). Collectively, these data are in agreement with the HCT116 findings and establish *USP22* as a CIN gene in two independent tissue- and cell-type contexts.

### 2.5. HCT116 USP22-KO Clones Exhibit Dynamic CIN Phenotypes over Time

**Figure 6:**
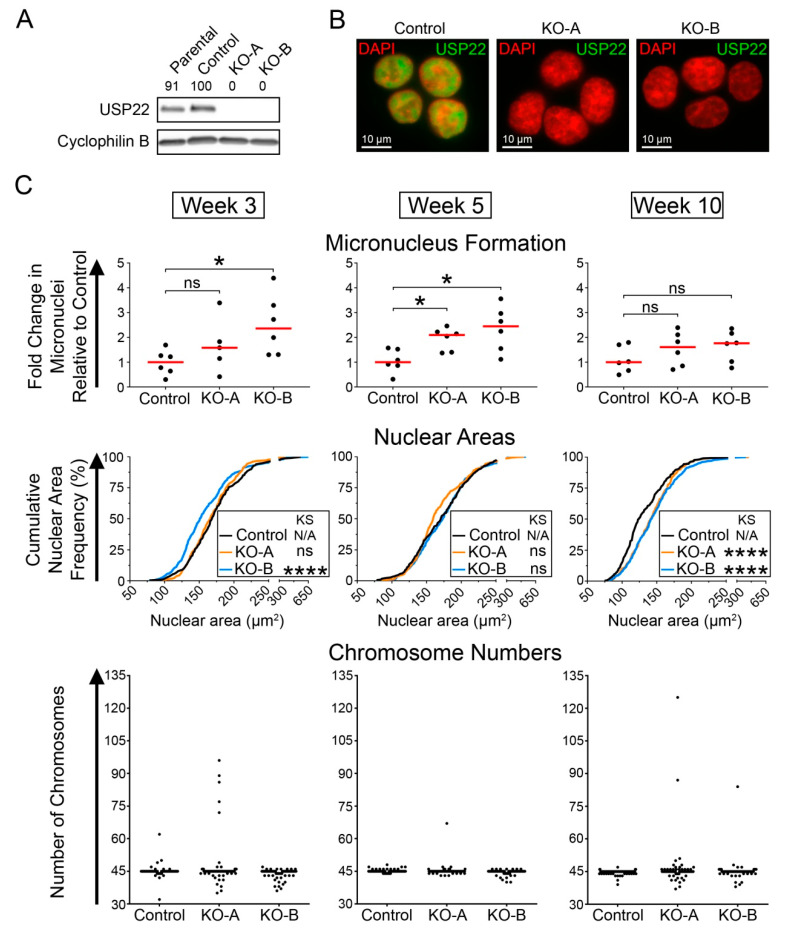
*USP22*-KO cells exhibit dynamic changes in nuclear areas, micronucleus formation and chromosome numbers. (**A**) Western blot presenting *USP22* expression levels in Parental HCT116, Control and *USP22*-KO-A and -B; cyclophilin B serves as the loading control. Semi-quantitative analyses were performed and the normalized USP22 levels are presented relative to the Control (100%). (**B**) High-resolution images of Control, KO-A and KO-B cells, immunofluorescently labeled for USP22 and counterstained with DAPI. For quantitative comparisons, all images were acquired using identical exposure times. (**C**) Changes in CIN phenotypes in *USP22*-KOs at three distinct time points (indicated at top). Dot plots presenting the fold change in micronucleus formation relative to the median of the Control at the indicated time points (top row). Mann–Whitney tests reveal significant changes in micronucleus formation relative to the Control (minimum 100 nuclei analyzed/replicate; ns *p*-value > 0.05; * *p*-value < 0.05). Cumulative nuclear area distribution frequencies of Control, *USP22*-KO-A and *USP22*-KO-B (middle row) reveal dynamic changes in nuclear areas in the *USP22*-KO clones relative to Control over time (300 nuclei analyzed/replicate; N/A not applicable; ns *p*-value > 0.01; **** *p*-value < 0.0001). Dot plot (bottom row) presenting the number of chromosomes enumerated from 100 mitotic chromosome spreads/condition.

Having established that reduced *USP22* expression induces CIN in short-term (<1 week) siRNA-based experiments, we now sought to determine the impact long-term USP22 depletion has on CIN. Using CRISPR-Cas9 approaches, two independent *USP22*-KO clones (KO-A and KO-B) and a Cas9 control were generated in HCT116 cells that were validated by Western blot, indirect immunofluorescence and DNA sequencing (Figure 6A,B and Figure S6). The *USP22*-KO clones and control cells were propagated for 10 weeks and CIN phenotypes were assessed at regular intervals (Figure 6C). In general, the *USP22*-KO clones exhibited a 1.6- to 2.4-fold increase in micronucleus formation relative to the control at each time point (Figure 6C; Table S7) and increases were greatest in KO-B, which displayed a significant 2.4-fold increase in median micronucleus formation at weeks 3 and 5. The dynamic changes in the abundance of micronuclei over the course of the experiment are consistent with ongoing changes in cell-to-cell heterogeneity induced by CIN. Each KO clone also exhibited dynamic changes in nuclear area distributions relative to the control (Figure 6C; Table S8). For example, although KO-A displayed a nuclear area distribution similar to that of the control at weeks 3 and 5, the cumulative nuclear area distribution frequency shifted dramatically towards larger nuclei at week 10. With respect to KO-B, the distributions were generally smaller at week 3 relative to the control, similar at week 5 and strikingly increased at week 10. In agreement with these temporal dynamics, mitotic spreads revealed continually evolving chromosome complements in both clones (Figure 6C; Table S9). More specifically, the frequency of abnormal chromosome numbers increased at week 3 in KO-A (32%) and KO-B (38%) relative to the control (22%) and decreased at week 5 (KO-A, 21%; KO-B, 29%), but increased again at week 10 (KO-A, 39%; KO-B, 30%; control, 24%). The dynamic changes in nuclear areas and chromosome numbers within the *USP22*-KO clones are in agreement with CIN, as ongoing chromosome mis-segregation events generate heterogeneous chromosome complements that confer different growth advantages and disadvantages, resulting in the selection of distinct cell populations harboring either losses or gains of chromosomes that are associated with smaller or larger nuclear areas, respectively. Collectively, these data show that *USP22*-KO induces dynamic changes in CIN-associated phenotypes and chromosome complements that are synonymous with CIN.

### 2.6. USP22 Deletions are Frequent in Cancer and Associated with Worse Patient Survival

**Figure 7:**
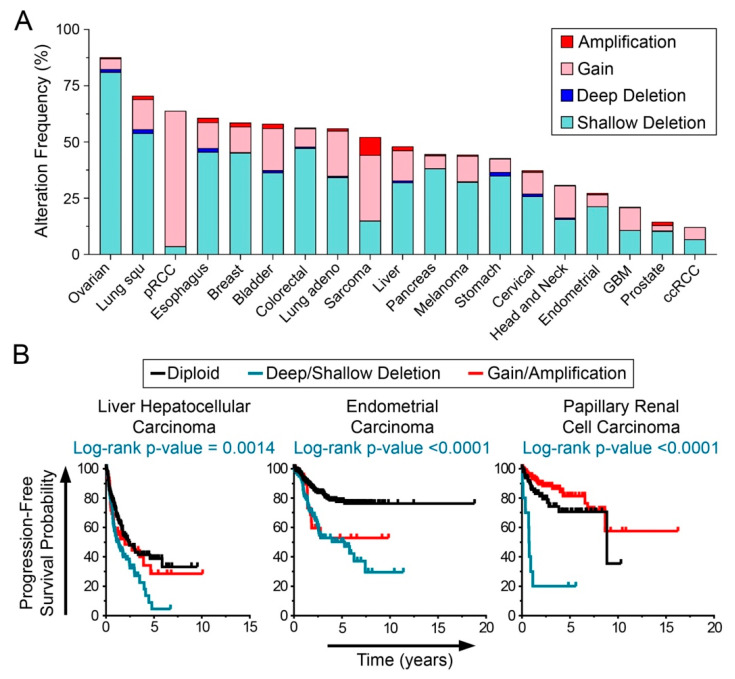
*USP22* deletions occur frequently in cancer. (**A**) Frequency of *USP22* alterations (shallow deletions, deep deletions (i.e., homozygous loss), gains and amplifications) in 19 cancer types [68,69]. Lung squ: lung squamous cell carcinoma; pRCC: papillary renal cell carcinoma; Lung adeno: lung adenocarcinoma; GBM: glioblastoma multiforme; ccRCC: clear cell renal cell carcinoma. Note that *USP22* is deleted (deep/shallow) in 10–80% of most cancers. (**B**) Kaplan–Meier curves comparing patient progression-free survival based on *USP22* copy number status (i.e., diploid, deep/shallow deletions or gain/amplification) reveal that *USP22* deletions correlate with worse patient survival in multiple cancer types. Log-rank *p*-values compare outcomes between diploid and deep/shallow deletions.

CIN is an aberrant phenotype observed in virtually all cancer types that is proposed to promote disease initiation and progression by increasing the rate at which key cancer genes (e.g., oncogenes; tumor suppressor; apoptotic; DNA repair genes) are gained, lost or altered [3,6,7,8]. Accordingly, CIN is often associated with aggressive tumors and poor patient prognosis [2,17,18]. To determine the potential clinical impact reduced *USP22* expression may have in cancer, The Cancer Genome Atlas (TCGA) data from 19 common cancer types were scrutinized (Figure 7A) and revealed that *USP22* copy number alterations occur frequently in multiple cancer types, ranging from 12% in renal clear cell carcinoma to 87% in ovarian cancer. Interestingly, deletions are more frequently observed than gains/amplifications in 17 of 19 cancer types evaluated, with shallow deletions being the most frequent, suggesting that deep/shallow deletions may be pathogenic events adversely impacting patient outcomes. To assess this possibility, TCGA patient data were stratified based on *USP22* copy number status and Kaplan–Meier survival plots (Figure 7B) determined that deep/shallow *USP22* deletions are associated with significantly reduced progression-free survival relative to diploid copy number status in several cancer types, including liver hepatocellular carcinoma (5-year progression-free survival 5% with *USP22* deletion vs. 38% with diploid status), endometrial cancer (50% vs. 77%) and papillary renal cell carcinoma (20% vs. 71%). Collectively, these patient-derived data are in agreement with *USP22* deletion and diminished expression being pathogenic events driving cancer progression via aberrant H2Bub1 regulation and CIN in multiple cancer types.

## 3. Discussion

In this study, we examined the functional impact reduced *USP22* expression (i.e., H2Bub1 abundance) has on chromosome structure and mitotic fidelity and gained clinical insight into its potential implications in cancer. Using complementary genetic and QuantIM approaches, we show that *USP22* silencing impairs H2Bub1 removal from chromosomes during prophase and correlates with increases in multiple CIN phenotypes. More specifically, *USP22* silencing induced significant increases in micronucleus formation, and changes in nuclear areas and chromosome complements in two distinct cellular contexts. To gain mechanistic insight, we determined that the timely removal of H2Bub1 is essential for proper chromatin compaction and sister kinetochore bi-orientation, which are required for the accurate segregation of chromosomes into daughter cells. To assess the long-term impact of USP22 depletion, two *USP22*-KO clones were newly generated and evaluated over a three-month timeframe. In agreement with the dynamic nature of CIN, both clones exhibited ongoing changes in micronucleus formation, nuclear areas and chromosome numbers over time. To determine the clinical implications of *USP22* copy number losses, TCGA data from 19 distinct cancer types were interrogated and it was determined that *USP22* is more frequently lost than gained in the majority (17/19) of cancers and that *USP22* copy number losses correspond with worse progression-free survival. Collectively, our findings reveal that the timely removal of H2Bub1 by USP22 in early mitotic stages is required to ensure mitotic fidelity and maintain chromosome stability and therefore identify *USP22* as a novel CIN gene. Thus, our fundamental and clinical findings strongly support the possibility that reduced *USP22* expression, leading to aberrant H2Bub1 regulation and CIN, are pathogenic events in many cancer types.

The failure to remove H2Bub1 following *USP22* silencing is associated with chromatin compaction defects that adversely impact mitotic fidelity potentially through multiple aberrant processes. For example, compaction defects have previously been shown to prevent sister chromatid disentanglement, resulting in a high frequency of anaphase bridges and chromosome mis-segregation events [65,70,71,72]. Chromatin compaction also imparts physical stiffness to the mitotic chromosomes, which counteracts the pulling forces exerted by the mitotic spindle to generate inter-kinetochore tension between sister chromatids [50,51,52]. Importantly, inter-kinetochore tension exhibits a critical role in the detection and correction of erroneous kinetochore–microtubule attachments (reviewed in [53]). In the present study, *USP22* silencing induced chromatin compaction defects in prometaphase and metaphase that were accompanied by enhanced kinetochore recruitment of the tension sensor BUB1 in metaphase. While increased BUB1 recruitment is indicative of inter-kinetochore tension defects within USP22-depleted cells, we did not observe a prolonged delay of anaphase onset, suggesting that BUB1 increases are either transient or insufficient to maintain SAC activation. In addition, we did not identify increases in chromosome congression defects or anaphase bridges but did observe increases in lagging chromosomes in anaphase. Collectively, these data imply that reduced *USP22* expression does not cause extensive compaction defects sufficient to prevent sister chromatid disentanglements and chromosome congression. Rather, reduced expression induces compaction defects that likely adversely impact chromatin stiffness, resulting in impaired inter-kinetochore tension that may compromise the correction of aberrant kinetochore–microtubule attachments and induce lagging chromosomes. This is supported by the findings of Ricke and colleagues [73], who determined that moderate increases in BUB1 abundance during mitosis induced increases in lagging chromosomes and changes in chromosome numbers that were not associated with increases in chromosome congression errors, anaphase bridges or delayed anaphase onset. Thus, it remains possible that USP22 depletion and/or moderate BUB1 overexpression selectively increase the frequency of lagging chromosomes by promoting merotelic kinetochore–microtubule attachments, an aberrant state that does not activate the SAC, to promote lagging chromosomes in anaphase without the formation of anaphase bridges. In addition, the mechanisms that correct merotelic attachments are expected to be particularly sensitive to subtle changes in inter-centromeric tension induced by changes in kinetochore microtubule occupancy [53], and therefore tension defects induced by aberrant chromatin compaction in USP22-depleted cells are likely to impair merotelic error correction. While this possibility remains to be formally evaluated, it is supported by the findings of Harasymiw and colleagues [57], who determined that altered chromatin stiffness selectively promotes lagging chromosomes but not chromatin bridges in anaphase. Although not all lagging chromosomes are expected to induce chromosome segregation errors, an increase in their abundance is predicted to enhance the probability of chromosome mis-segregation events leading to CIN. However, as H2Bub1 also impacts additional biological processes, such as transcription [35,36] and DNA damage repair [37,38,39], it remains possible that additional mechanisms may also contribute to the CIN phenotypes. In particular, the removal of H2Bub1 by USP22 is critical for DNA double-strand break (DSB) repair by both non-homologous end joining and homologous recombination repair pathways in mice [46,48], while emerging data indicate that USP22 is also required for DSB repair in humans [74]. Conceivably, reduced *USP22* expression inducing DSB repair defects may lead to chromosomal rearrangements in addition to the numerical chromosome changes identified in this study. For instance, DSB repair defects may produce acentric chromosome fragments (i.e., lacking a functional kinetochore) that fail to properly segregate, resulting in micronucleus formation [75]; however, while chromosomal rearrangements were not formally assessed within USP22-depleted cells, it should be noted that we did not readily observe increases in chromosome fragments (i.e., indicative of DSBs) within the mitotic chromosome spreads of USP22-depleted cells, suggesting that *USP22* deletion may not induce frequent rearrangements. Finally, it should be highlighted that USP22 also targets additional substrates beyond H2Bub1, including SIRT1, FBP1 and Cyclin D1 (reviewed in [76]), and thus it will be important to establish whether they also impact CIN.

A recent and growing body of evidence supports the possibility that distinct levels of CIN may differentially impact cancer initiation, progression and patient outcomes. Notably, data from mathematical modeling [12], mammalian cell lines [9,11,77,78] and mouse models [10,79] implicate intermediate levels as a dominant driver of tumor initiation and development, as high levels are often associated with catastrophic events that decrease cell fitness and viability, while intermediate levels typically correspond with greater cell fitness/viability. For example, while heterozygous loss of either *Mad2* or *Cenpe* (mitotic checkpoint genes) induces intermediate CIN levels that promote spontaneous tumor formation in mice, simultaneous loss induces high levels that correspond with reduced tumor formation [10]. In further support of the threshold concept, intermediate rather than high levels of CIN correlate with worse patient outcomes in a number of cancer types, including ovarian, gastric, non-small cell lung and estrogen receptor-negative breast cancer patients [80,81]. Collectively, these data highlight the importance of identifying not only the molecular determinants of CIN, but especially those that induce intermediate levels, as these are more likely to be associated with cancer pathogenesis. In this regard, the present study identified intermediate CIN phenotypes following reduced *USP22* expression, relative to the large-scale changes associated with other CIN genes such as *KIF11*, cohesion and condensin genes assessed using similar QuantIM approaches in HCT116 and hTERT [21,65,67]. This observation suggests that the global increases in H2Bub1 observed in several cancers [82,83] may be a novel mechanism underlying intermediate CIN levels that contribute to worse patient outcomes. This possibility is further buttressed by patient survival curves (Figure 7B) showing that *USP22* deletions correspond with worse survival in numerous cancer types. Accordingly, future studies aimed at identifying additional pathways driving intermediate CIN levels will be critical to develop effective precision medicine strategies (i.e., synthetic lethality) that exploit these aberrant features to ultimately minimize the morbidity and mortality rates associated with cancer.

While recent TCGA data reveal that deep/shallow *USP22* deletions are more prevalent than gain/amplification and that *USP22* expression is frequently reduced at the mRNA level in most cancer types [68,69,84], increased *USP22* expression has also been reported in multiple cancers and *USP22* has traditionally been investigated as an oncogene [85,86,87,88,89,90,91,92]. Interestingly, *USP22* deletions are associated with reduced survival in several cancer types, suggesting that reduced expression/function may also contribute to cancer initiation and progression. In agreement with this possibility, we show that diminished *USP22* expression induces CIN, highlighting a novel role for *USP22* as a tumor suppressor that is essential to maintain mitotic fidelity and chromosome stability. In this regard, while USP22 has been proposed as a novel therapeutic target based on its oncogenic functions [93,94,95], our work suggests that USP22 inhibition will induce CIN that may promote cancer progression and/or the development of secondary malignancies. Moreover, our findings may contribute to recent results by Kosinsky and colleagues [96] identifying a tumor suppressor function for *USP22* within a colorectal cancer context. Thus, our findings coupled with emerging data caution against the use of USP22 inhibitors and highlight the need for greater insight into the impact on oncogenesis of misregulation of USP22 and its substrate H2Bub1.

## 4. Materials and Methods

### 4.1. Cell Lines and Culture

HCT116 (male human colorectal carcinoma) cells were purchased from the American Type Culture Collection (Rockville, MD, USA), while the immortalized (human telomerase) hTERT (male normal skin fibroblast) cells were provided by Dr. C.P. Case (University of Bristol, Bristol, UK). Cell lines were authenticated on the basis of growth, morphology and spectral karyotyping [97]. HCT116 and hTERT were grown in McCoy’s 5A (Hyclone) and DMEM (Hyclone) media, respectively, and supplemented with 10% fetal bovine serum. All cells were maintained in a 37 °C humidified incubator containing 5% CO_2_.

### 4.2. Gene Silencing and Western Blot

ON-TARGET*plus* siRNA duplexes (Dharmacon) were employed either as individual siRNA duplexes (siUSP22-1, -2, -3 or -4) targeting distinct coding regions of the targeted mRNA, or as a pool (siUSP22-Pool) comprised of equimolar amounts of the four individual siRNAs. A negative control siRNA (siControl) was employed in all silencing experiments. Cells were transfected using RNAiMAX (ThermoFisher Scientific) according to the manufacturer’s instructions. Gene silencing was confirmed by Western blot as detailed elsewhere [21,98] using the antibodies and dilutions indicated in Table S10. Semi-quantitative Western blot analysis was performed with ImageJ software, where the protein of interest was normalized to the respective loading control (cyclophilin B or α-tubulin) and presented relative to siControl (100%; Figures S7 and S8). To assess H2Bub1 abundance, acid-based histone extractions were performed as described elsewhere [99] and the soluble and histone fractions were analyzed by Western blot to assess *USP22* silencing efficiency and H2Bub1 abundance, respectively. H2B was employed as loading control for the histone fraction and semi-quantitative Western blot analysis was performed as described above. Figures were assembled in Photoshop CS6 (Adobe).

### 4.3. Indirect Immunofluorescence

Indirect immunofluorescence labeling of USP22, H2Bub1, PhosS10, ACA and BUB1 was performed as described elsewhere [100], with the antibodies and dilutions listed in Table S10. Briefly, asynchronous cells were paraformaldehyde-fixed (4%), immunofluorescently labeled and counterstained with DAPI. For H2Bub1 or BUB1 labeling, asynchronous HCT116 cells were fixed 48 h post-transfection, with each experiment conducted a total of two times. For H2Bub1 labeling, an antigen retrieval step was performed as described previously [40].

### 4.4. CRISPR-Cas9 Gene Editing

HCT116 *USP22*-KO cells were generated with the Edit-R CRISPR-Cas9 Gene Engineering platform according to the manufacturer (Dharmacon). Briefly, the Edit-R Cas9 expression vector was transduced (lentiviral) into HCT116 cells and clonally selected. Next, a crRNA targeting exon 3 of *USP22* (5′-CUUUGUCAUAGAUGUAGUCC) was designed with the CRISPR-Cas9 sgRNA design tool (http://crispr.mit.edu, accessed on 1 August 2015) [101], synthesized (Dharmacon), complexed with tracrRNA and transfected into the Cas9-expressing cell line. Two transfectants were clonally expanded and *USP22*-KO was confirmed by Western blot, indirect immunofluorescence and DNA sequencing (Genome Quebec; Figure S6). The control cell line was generated by transfecting tracrRNA without crRNA into the Cas9-expressing cell line (mock transfection).

### 4.5. QuantIM

Changes in nuclear area and micronucleus formation were quantitatively assessed as described elsewhere [61,62]. Briefly, cells were paraformaldehyde-fixed and counterstained with Hoechst 33342 (DNA/nuclear marker). QuantIM was performed using a Cytation 3 Cell Imaging Multi-mode Reader (BioTek) equipped with a 16-bit CCD camera and a 20× Olympus lens (0.45 numerical aperture). Nine overlapping images (3 × 3 matrix)/well were acquired from a 96-well plate and stitched together with Gen5 software (BioTek). Each condition was performed in sextuplet and experiments were conducted a total of two times. Nuclear areas and micronucleus formation were quantified using Gen5 software, with previously defined inclusion and exclusion criteria [61,62]. Briefly, primary nuclei were distinguished from apoptotic bodies using a size-inclusion filter (>80 µm^2^), while micronuclei were defined as extra-nuclear Hoechst-stained bodies, with no visible attachment to the primary nuclei and with a size < 1/3 of the primary nucleus. Nuclear areas were imported into Prism software (GraphPad) and nuclear area distributions were compared with the two-sample KS test, and *p*-values < 0.01 were considered significant. Micronuclei counts were normalized to the number of primary nuclei and expressed as fold change in micronucleus formation relative to the mean of the control. Results were compared using Mann–Whitney tests, and *p*-values < 0.05 were considered significant.

Semi-quantitative fluorescence imaging microscopy analysis of H2Bub1 abundance was performed by collecting three-dimensional images with an AxioImager Z1 microscope (Zeiss) equipped with an AxioCam HRm CCD camera and a 63× oil immersion Plan-Apochromat lens (1.30 numerical aperture). Exposure times were first optimized for each channel and maintained constant throughout the entire image acquisition phase. Approximately 25 optical sections/image were acquired at 0.400 µm intervals using the DAPI, FITC and Cy3 filters to acquire nuclear, H2Bub1 and PhosS10 data, respectively. A minimum of 25 prophase cells/condition were imaged based on standard cytological criteria (i.e., prior to nuclear envelope breakdown). Images were imported into Imaris v7.7.1 software (Bitplane) where 3D renderings of the prophase nuclei were generated based on PhosS10 labeling, and mean H2Bub1 signal intensities were determined for each prophase nucleus and imported into Prism software for statistical analysis. Conditions were compared using Mann–Whitney tests, and *p*-values < 0.05 were considered significant. BUB1 signal intensities were quantified in an analogous fashion with the following modifications: (1) DAPI, FITC and Cy3 channels were employed to acquire nuclear, ACA and BUB1 data, respectively; (2) a minimum of 40 images were acquired per silencing condition; and (3) a minimum of 10 images were acquired for the negative (DMSO; vehicle control) and positive (Taxol; 10 µM for 30 min) controls. To assess BUB1 abundance at kinetochores, images were deconvolved in AutoQuant X3 (Media Cybernetics) and imported into Imaris where an intensity threshold mask was applied to the Cy3 (BUB1) channel to quantify individual kinetochore-associated BUB1 foci. To assess BUB1 kinetochore recruitment, average BUB1 focal volumes and intensities were determined for each metaphase cell. In addition, signal intensities from each focus were added up to calculate the total kinetochore-associated BUB1 signal for each metaphase cell. Conditions were compared using Mann–Whitney tests, and *p*-values < 0.05 were considered significant.

To quantify aberrant mitotic events following silencing, asynchronous cells were seeded onto coverslips, paraformaldehyde-fixed 48 h post-transfection, immunofluorescently labeled (PhosS10) and counterstained (DAPI). Metaphase and anaphase cells were imaged using an AxioImager Z1 microscope and were classified into normal or aberrant mitotic categories based on classical cytological features.

### 4.6. Live-Cell Imaging

To assess mitotic progression, HCT116 cells stably expressing H2B-GFP were silenced as described above (Section 4.2) and cells were imaged every 2 min 30 s for 45 min using the Cytation 3 Reader equipped with a 20× Olympus lens (0.45 numerical aperture). Exposure times were optimized to minimize phototoxicity. The time from nuclear envelope breakdown to anaphase entry was determined using standard cytological criteria for a minimum of 25 mitotic cells/condition.

### 4.7. Preparation and Assessment of Mitotic Chromosome Spreads

To allow for equal numbers (~4) of cell doublings post-transfection, HCT116 cells were permitted to grow for 96 h and hTERT cells for 144 h, at which point mitotic spreads were generated as described [102]. Briefly, subconfluent cells were mitotically enriched with KaryoMAX colcemid (100 ng/mL, Gibco) for 2 (HCT116) or 4 h (hTERT), treated with hypotonic solution for 16 (HCT116) or 12 min (hTERT) and fixed with three 10 min washes of methanol/acetic acid (3:1). Chromosomes were counterstained (DAPI) and spreads were imaged using an AxioImager Z2 microscope (Zeiss) equipped with an AxioCam HRm CCD camera and a 63× oil immersion Plan-Apochromat lens (1.40 numerical aperture). Then, 16-bit TIFF images were acquired and imported into ImageJ software, where chromosomes were manually enumerated from 100 spreads/condition, with each experiment repeated three times. The average frequencies of abnormal spreads were compared with Student’s *t*-tests, and *p*-values < 0.05 were considered significant.

### 4.8. Super-Resolution STED Microscopy

HCT116 cells were seeded onto high-performance cover glass (Carl Zeiss) where they were grown and silenced as detailed above). Cells were fixed 48 h post-transfection with filtered 4% paraformaldehyde, and DNA was counterstained using SiR-Hoechst (STED compatible [103]; Cytoskeleton, Inc; CY-SC007). Images were acquired with a Falcon SP8 microscope (Leica) equipped with a 100× oil immersion Plan-Apochromat objective (1.40 numerical aperture) and a 775 nm STED laser. The excitation laser was set to 635 nm and the signal was detected by an HyD detector set to a 650–700 nm interval with 0.3–9.9 ns time gating. The pinhole was set to 1 a.u. and images were acquired using 16 times line averaging, 2 frame accumulations and a pixel size in the xy plane of 28.41 × 28.41 nm.

### 4.9. Analysis of USP22 Alterations and Impact on Survival in Cancer

All DNA sequencing and copy number status data were acquired from TCGA (Pan-Cancer atlas data) [68,69]. Survival data were downloaded from TCGA, stratified by *USP22* copy number status and imported into Prism software for statistical analysis. Survival curves associated with shallow/deep deletion or diploid status were statistically compared, and a log-rank *p*-value < 0.05 was considered significant.

## 5. Conclusions

In summary, these data show that precise regulation of H2Bub1 dynamics in mitosis is critical to achieve higher-order compaction and maintain faithful chromosome segregation into daughter cells. In addition, we reveal that *USP22* deficiency impairs H2Bub1 removal in early mitosis and induces CIN. As *USP22* deletions occur frequently and are associated with reduced patient survival in multiple cancer types, this study indicates that reduced *USP22* expression and aberrant H2Bub1 regulation in tumors may drive genetic heterogeneity and promote cancer pathogenesis. These findings can support the development of novel synthetic lethality-based therapeutic approaches that exploit *USP22* deficiency to improve the outcomes of cancer patients whose tumors exhibit reduced *USP22* expression, within a precision medicine framework.

## Figures and Tables

**Table 1:** *USP22* silencing increases the frequency of lagging chromosomes in HCT116.

Mitotic Stage	Category	siControl		siUSP22-Pool		
		Percentage	Number of Cells	Percentage	Number of Cells	Fold Change ^A^
Metaphase	Normal	86.5%	135	87.9%	218	1.02
	Congression defects	13.5%	21	12.1%	30	0.90
Anaphase	Normal	80.0%	136	73.2%	134	0.91
	Chromatin bridges	17.1%	29	16.4%	30	0.96
	Lagging chromosomes	2.9%	5	10.4%	19	3.59

^A^ Fold change in the frequency of the phenotype relative to siControl.

## Data Availability

All descriptive statistics and statistical analyses presented in Figure 1, Figure 2, Figure 3, Figure 4, Figure 5 and Figure 6 are provided within Supplementary Tables S1–S9. Patient-related data (Figure 7) are based upon data generated by the TCGA Research Network and are available at https://www.cancer.gov/tcga, accessed on 12 September 2020.

## Supplementary Materials

The following are available online at https://www.mdpi.com/2072-6694/13/5/1043/s1, Figure S1: *USP22* silencing increases H2Bub1 abundance in asynchronous HCT116 cells; Figure S2: Reduced *USP22* expression corresponds with increases in chromosome segregation errors; Figure S3: *USP22* silencing increases BUB1 recruitment to kinetochores in HCT116 metaphase cells; Figure S4: *USP22* silencing is not associated with a prolonged delay of anaphase onset; Figure S5: *USP22* silencing is not associated with reproducible increases in micronucleus formation in hTERT cells; Figure S6: DNA sequencing identifies two homozygous **USP22**-KO clones in HCT116; Figure S7: Raw data for Western blots in Figure 1 and Figure S1. Figure S8: Raw data for Western blots in Figure 5 and Figure 6; Table S1: **USP22** silencing corresponds with global increases in H2Bub1 levels within prophase HCT116 cells; Table S2: **USP22** silencing increases the frequency of chromatin compaction defects within mitotic HCT116 cells; Table S3: *USP22* silencing induces significant increases in BUB1 recruitment to kinetochores within metaphase HCT116 cells; Table S4: Mann–Whitney tests reveal significant increases in micronucleus formation following *USP22* silencing in HCT116 cells; Table S5: KS tests identify statistically significant changes in nuclear areas following *USP22* silencing; Table S6: *USP22* silencing induces numerical changes in chromosome complements; Table S7: Mann–Whitney tests reveal significant changes in micronucleus formation in *USP22*-KO clones; Table S8: KS tests reveal significant changes in nuclear areas in *USP22*-KO clones; Table S9: *USP22*-KO clones exhibit dynamic changes in chromosome numbers; Table S10: Antibody sources and dilutions.
