# Supplementary material for: Reduced USP22 Expression Impairs Mitotic Removal of H2B Monoubiquitination, Alters Chromatin Compaction and Induces Chromosome Instability That May Promote Oncogenesis
Source: Cancers (Basel). 2021 Mar 2;13(5):1043. doi: 10.3390/cancers13051043 (PMC7958346; doi:10.3390/cancers13051043)
Supplement: Supplementary file 1 [file cancers-13-01043-s001.pdf]

cancers

## Supplementary Figures

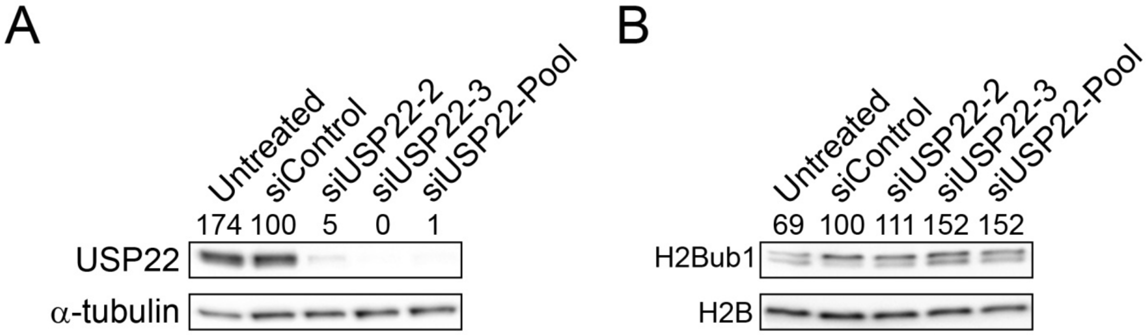


**Figure S1.** *USP22* silencing increases H2Bub1 abundance in asynchronous HCT116 cells. **(A)** Western blot presenting USP22 abundance following silencing in HCT116 with the best two individual (siUSP22-2, -3) or pooled *USP22* (siUSP22-Pool) siRNAs and controls (untreated or siControl). Protein samples analyzed correspond to the soluble (i.e. non-histone) fraction of the histone extraction samples displayed in (B). $\alpha$-tubulin serves as the loading control. Semi-quantitative analyses were performed and the normalized USP22 levels are presented relative to siControl (100%). **(B)** Western blot presenting H2Bub1 abundance following silencing in HCT116; H2B serves as the loading control. Semi-quantitative analyses were performed and the normalized H2Bub1 levels are presented relative to siControl (100%).

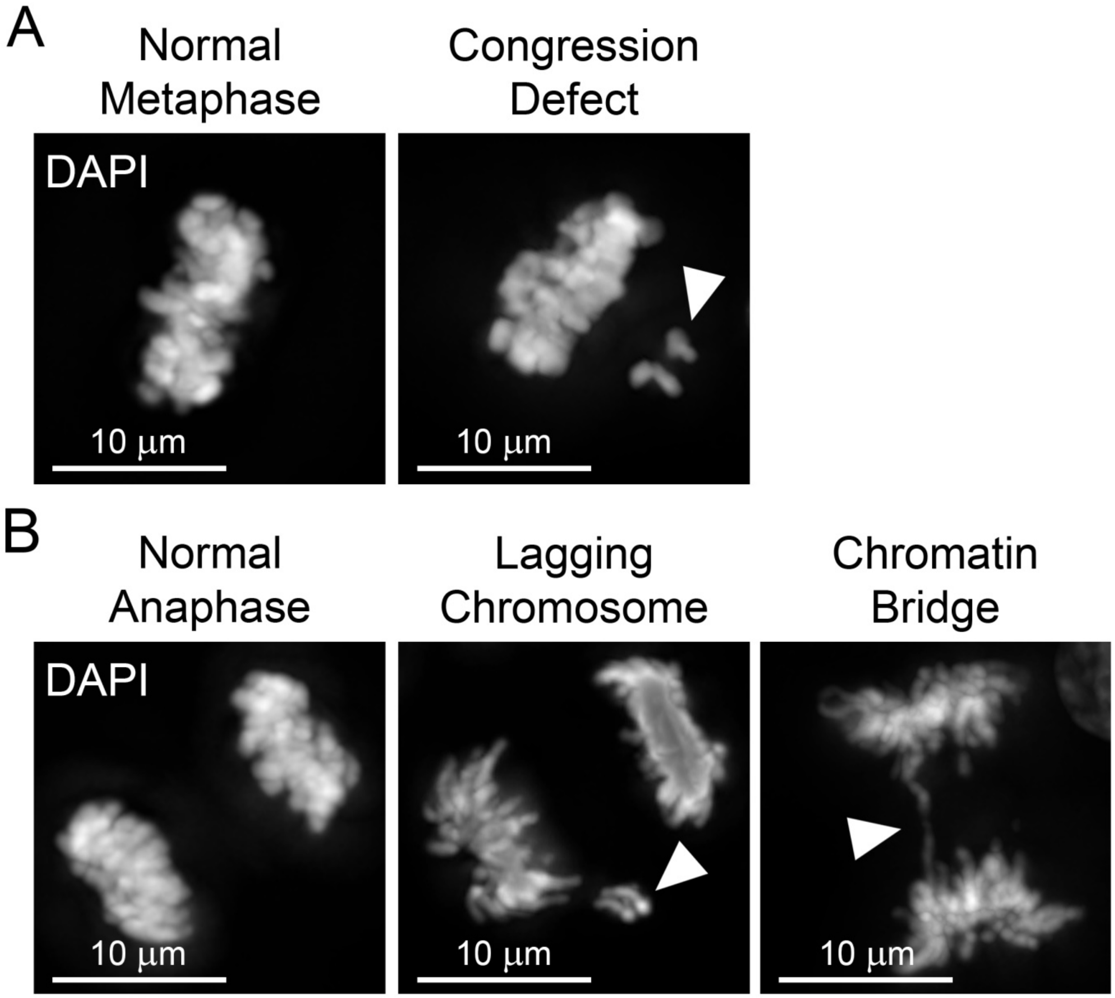


**Figure S2.** Reduced *USP22* expression corresponds with increases in chromosome segregation errors. **(A)** Representative maximum intensity projections of a normal metaphase cell (left) and an abnormal metaphase cell (right) exhibiting a chromosome congression error (arrowhead). **(B)** Representative maximum intensity projections of a normal anaphase cell (left) and abnormal anaphase cells exhibiting a segregation error (middle; lagging chromosomes; arrowhead) and a chromatin bridge (right; arrowhead).

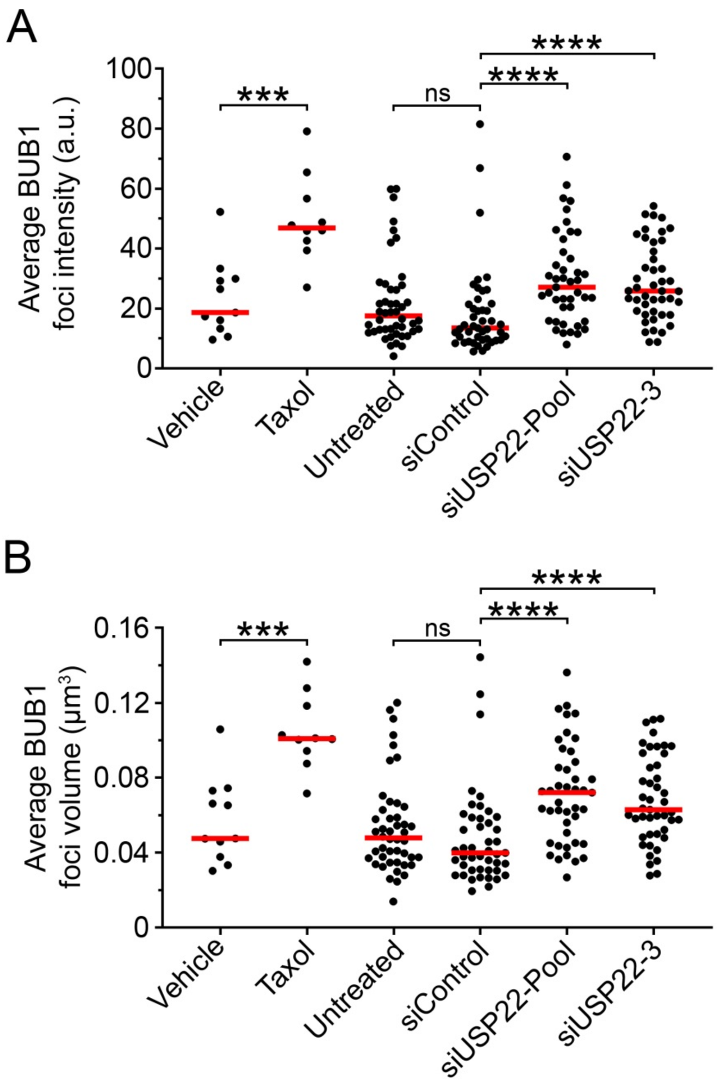


**Figure S3.** *USP22* silencing increases BUB1 recruitment to kinetochores in HCT116 metaphase cells. **(A)** Dot plot presenting the average kinetochore-associated BUB1 focus intensity/cell, with red bars indicating median intensities. Mann-Whitney tests reveal significant increases in the rank order of BUB1 intensities (i.e. levels) following Taxol treatment (positive control) relative to vehicle control and following *USP22* silencing relative to siControl (N = 2; $n \geq 10$ for vehicle and Taxol controls; $n > 40$ for silencing conditions; ns $p$-value > 0.05; *** $p$-value < 0.001; **** $p$-value < 0.0001). **(B)** Dot plot showing the average BUB1 kinetochore volume, with red bars indicating the median focus volume. Mann-Whitney tests identify significant increases in BUB1 foci volumes following Taxol treatment (positive control) and following *USP22* silencing relative to siControl (N = 2; $n \geq 10$ for vehicle and Taxol controls; $n > 40$ for silencing conditions; ns $p$-value > 0.05; *** $p$-value < 0.001; **** $p$-value < 0.0001).

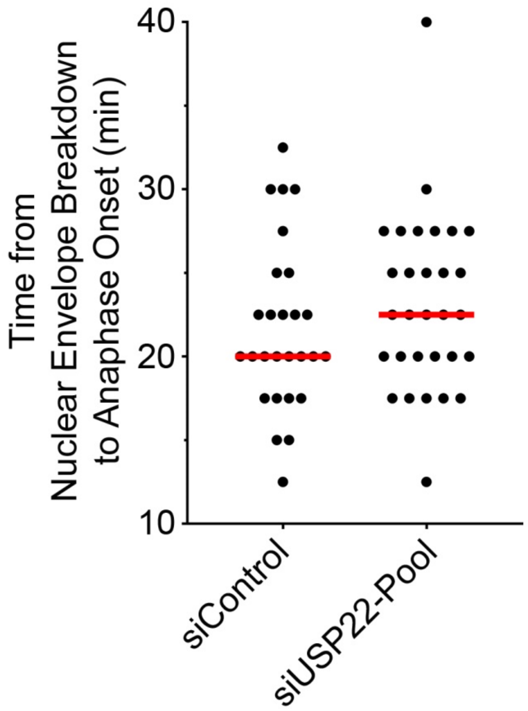


**Figure S4.** *USP22* silencing is not associated with a prolonged delay of anaphase onset**.** Dot plot presenting the time from nuclear envelope breakdown to anaphase onset following control or *USP22* silencing. Red bars indicate the median. A minimum of 25 mitotic cells/condition were monitored by live cell microscopy.

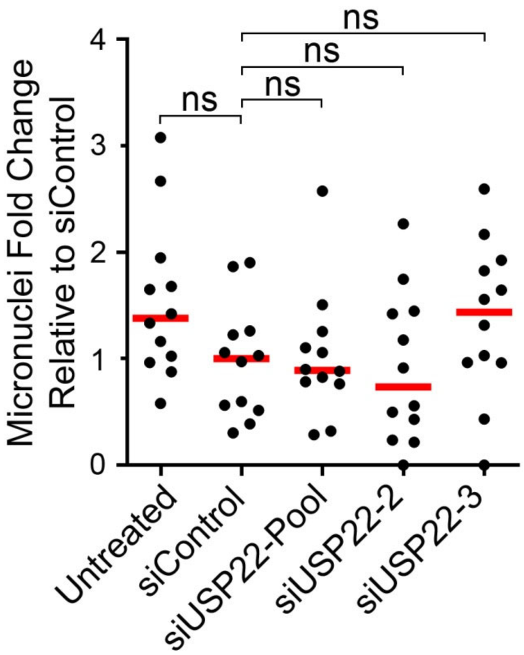


**Figure S5.** *USP22* silencing is not associated with reproducible increases in micronucleus formation in hTERT cells. Dot plot presenting the fold change in micronucleus formation relative to the median of siControl; red bars identify median values. Mann-Whitney tests did not identify statistically significant increases in micronucleus formation following *USP22* silencing relative to siControl (N = 2; $n$ = 12; minimum 100 nuclei analyzed/replicate; ns [not significant] $p$-value > 0.05).

**A**

```
                        Nucleotide 23 of exon 3                               Nucleotide 72 of exon 3
WT exon 3 reference   5'-ATCTACTGTTTTCTGTGCCAGGA-CTACATCTATGACAAAGACATGGAAAT-3'
USP22-KO-A Allele 1   5'-ATCTACTGTTTTCTGTGCCAGGACCTACATCTATGACAAAGACATGGAAAT-3'
USP22-KO-A Allele 2   5'-ATCTACTGTTTTCTGTGCCAGGA--TACATCTATGACAAAGACATGGAAAT-3'

WT exon 3 reference   5'-ATCTACTGTTTTCTGTGCCAGGAC----TACATCTATGACAAAGACATGGAAAT-3'
USP22-KO-B Allele 1   5'-ATCTACTGTTTTCTGTGCCAGGACTC--TACATCTATGACAAAGACATGGAAAT-3'
USP22-KO-B Allele 2   5'-ATCTACTGTTTTCTGTGCCAGAAAACAGTACATCTATGACAAAGACATGGAAAT-3'
```

**B**

```
WT protein            MVSRPEPEGEAMDAELAVAPPGCSHLGSFKVDNWKQNLRAIYQCFVWSGTAEARKRKAKS
USP22-KO-A Allele 1   MVSRPEPEGEAMDAELAVAPPGCSHLGSFKVDNWKQNLRAIYQCFVWSGTAEARKRKAKS
USP22-KO-A Allele 2   MVSRPEPEGEAMDAELAVAPPGCSHLGSFKVDNWKQNLRAIYQCFVWSGTAEARKRKAKS
USP22-KO-B Allele 1   MVSRPEPEGEAMDAELAVAPPGCSHLGSFKVDNWKQNLRAIYQCFVWSGTAEARKRKAKS
USP22-KO-B Allele 2   MVSRPEPEGEAMDAELAVAPPGCSHLGSFKVDNWKQNLRAIYQCFVWSGTAEARKRKAKS

WT protein            CICHVCGVHLNRLHSCLYCVFFGCFTKKHIHEHAKAKRHNLAIDLMYGGIYCFLCQDYIY
USP22-KO-A Allele 1   CICHVCGVHLNRLHSCLYCVFFGCFTKKHIHEHAKAKRHNLAIDLMYGGIYCFLCQDLHL
USP22-KO-A Allele 2   CICHVCGVHLNRLHSCLYCVFFGCFTKKHIHEHAKAKRHNLAIDLMYGGIYCFLCQDTSM
USP22-KO-B Allele 1   CICHVCGVHLNRLHSCLYCVFFGCFTKKHIHEHAKAKRHNLAIDLMYGGIYCFLCQDSTS
USP22-KO-B Allele 2   CICHVCGVHLNRLHSCLYCVFFGCFTKKHIHEHAKAKRHNLAIDLMYGGIYCFLCQKTVH

WT protein            DKDMEIIAKEEQRKAWKMQGVGEKFSTWEPTKRELELLKHNPKRRKITSNCTIGLRGLIN
USP22-KO-A Allele 1   *
USP22-KO-A Allele 2   TKTWK*
USP22-KO-B Allele 1   MTKTWK*
USP22-KO-B Allele 1   L*

WT protein            LGNTCFMNCIVQALTHTPLLRDFFLSDRHRCEMQSPSSCLVCEMSSLFQEFYSGHRSPHI

WT protein            PYKLLHLVWTHARHLAGYEQQDAHEFLIAALDVLHRHCKGDDNGKKANNPNHCNCIIDQI

WT protein            FTGGLQSDVTCQVCHGVSTTIDPFWDISLDLPGSSTPFWPLSPGSEGNVVNGESHVSGTT

WT protein            TLTDCLRRFTRPEHLGSSAKIKCSGCHSYQESTKQLTMKKLPIVACFHLKRFEHSAKLRR

WT protein            KITTYVSFPLELDMTPFMASSKESRMNGQYQQPTDSLNNDNKYSLFAVVNHQGTLESGHY

WT protein            TSFIRQHKDQWFKCDDAIITKASIKDVLDSEGYLLFYHKQFLEYE*
```

**Figure S6.** DNA sequencing identifies two homozygous *USP22*-KO clones in HCT116**. (A)** DNA sequences of *USP22* exon 3 in the wild-type control (WT; reference) and the two *USP22*-KO clones. The crRNA target sequence is indicated in bold within the reference sequence, while the protospacer adjacent motif (PAM) is underlined. **(B)** Protein sequences corresponding to the WT USP22 reference and the hypothetical proteins encoded by the CRISPR-Cas9 edited alleles in the *USP22*-KO clones. Alternating exons are highlighted in black and blue. Red amino acids are encoded by codons located at an exon-exon junction, while * identifies a stop codon. Aberrant amino acids encoded by the edited alleles are indicated in bold. Note that the edited alleles all encode a premature stop codon located over 50 bp upstream of the last exon-exon junction, which targets the corresponding mRNA for non-sense mediated decay and precludes the translation of the hypothetical aberrant proteins.

A

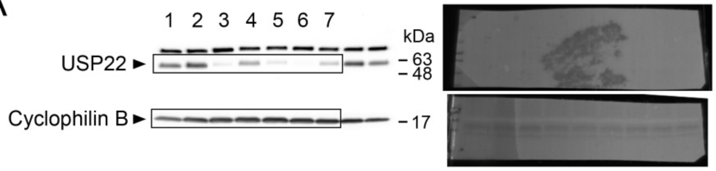


| | USP22 | Cyclophilin B | Ratio U/C | Norm. ratio |
|---|---|---|---|---|
| **Lane 1** | 70.715 | 36.436 | 1.940 | 94.171 |
| **Lane 2** | 96.643 | 46.893 | 2.060 | 100.000 |
| **Lane 3** | 8.926 | 51.674 | 0.172 | 8.381 |
| **Lane 4** | 41.748 | 55.228 | 0.755 | 36.678 |
| **Lane 5** | 9.975 | 59.508 | 0.167 | 8.133 |
| **Lane 6** | 2.73 | 55.843 | 0.048 | 2.372 |
| **Lane 7** | 21.873 | 54.486 | 0.401 | 19.478 |

B

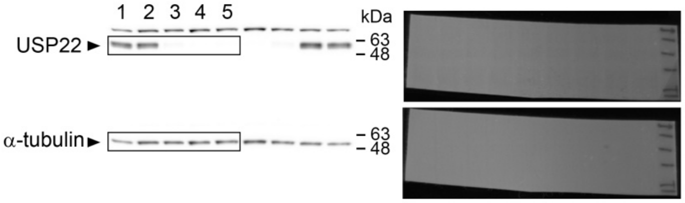


| | USP22 | α–tubulin | Ratio U/α | Norm. ratio |
|---|---|---|---|---|
| **Lane 1** | 81.870 | 28.271 | 2.8960 | 173.876 |
| **Lane 2** | 89.443 | 53.702 | 1.6655 | 100.000 |
| **Lane 3** | 3.526 | 46.101 | 0.0765 | 4.592 |
| **Lane 4** | 0.290 | 54.242 | 0.0054 | 0.321 |
| **Lane 5** | 0.806 | 51.216 | 0.0157 | 0.944 |

C

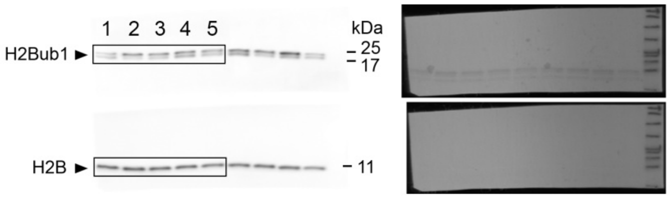


| | H2Bub1 | H2B | Ratio H2Bub1/H2B | Norm. ratio |
|---|---|---|---|---|
| **Lane 1** | 21.341 | 80.679 | 0.2645 | 69.045 |
| **Lane 2** | 34.780 | 90.783 | 0.3831 | 100.000 |
| **Lane 3** | 34.372 | 80.922 | 0.4248 | 110.872 |
| **Lane 4** | 45.523 | 77.884 | 0.5845 | 152.567 |
| **Lane 5** | 39.202 | 67.130 | 0.5840 | 152.430 |

**Figure S7.** Raw data for western blots in Figures 1 and S1**. (A)** Chemiluminescence (left) and visible light (right) images of the USP22 and Cyclophilin B western blots shown in Figure 1A. Black rectangles indicate cropped sections shown in Figure 1A. Densitometry analyses for USP22 and Cyclophilin-B were performed using Image J and are indicated. The ratio of USP22/Cyclophilin-B is shown for each lane, as are the normalized ratios, which are presented relative to the siControl (Lane 2). **(B)** Chemiluminescence (left) and visible light (right) images of the USP22 and $\alpha$-tubulin western blots shown in Figure S1A. Black rectangles indicate cropped sections shown in Figure S1A. Densitometry analyses for USP22 and $\alpha$-tubulin are indicated. The ratio of USP22/$\alpha$-tubulin is shown for each lane, as are the normalized ratios, which are presented relative to the siControl (Lane 2). **(C)** Chemiluminescence (left) and visible light (right) images of the H2Bub1 and H2B western blots shown in Figure S1B. Black rectangles indicate cropped sections shown in Figure S1B. Densitometry analyses for H2Bub1 and H2B are indicated. The ratio of H2Bub1/H2B is shown for each lane, as are the normalized ratios, which are presented relative to siControl (Lane 2).

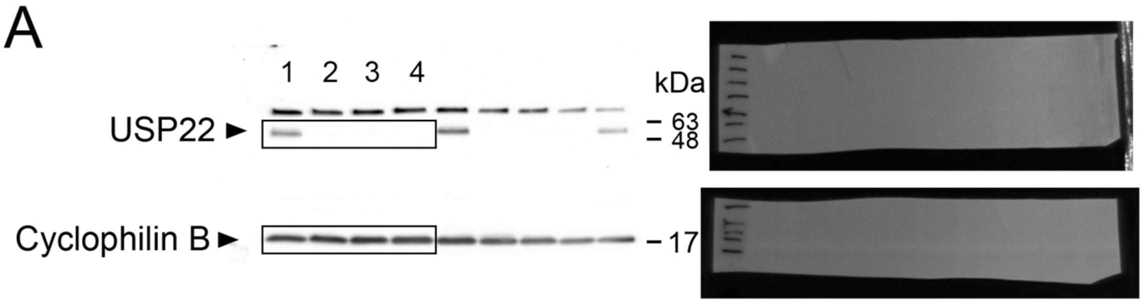


| | USP22 | Cyclophilin B | Ratio U/C | Norm. ratio |
|---|---|---|---|---|
| **Lane 1** | 51.079 | 94.441 | 0.540 | 100.000 |
| **Lane 2** | 0.796 | 104.253 | 0.007 | 1.411 |
| **Lane 3** | 1.029 | 107.138 | 0.009 | 1.775 |
| **Lane 4** | 0.291 | 107.239 | 0.002 | 0.501 |

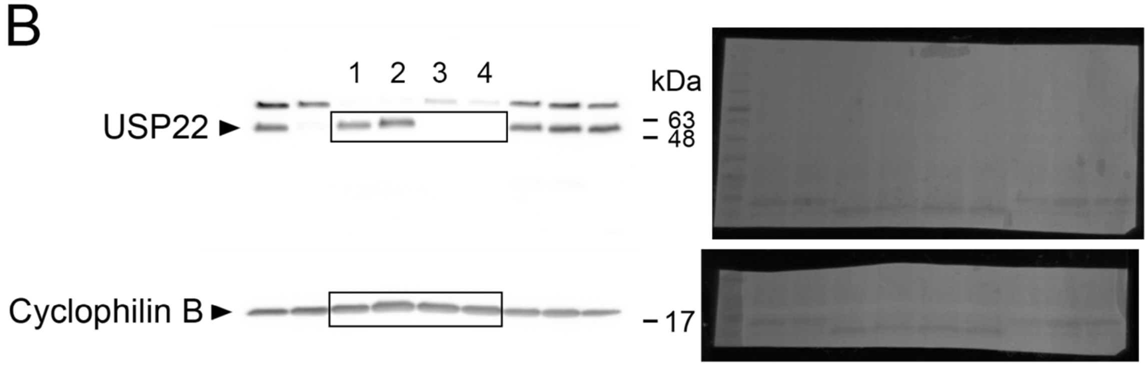


| | USP22 | Cyclophilin B | Ratio U/C | Norm. ratio |
|---|---|---|---|---|
| **Lane 1** | 47.677 | 58.190 | 0.819 | 90.853 |
| **Lane 2** | 65.335 | 72.448 | 0.901 | 100.000 |
| **Lane 3** | 0.000 | 64.921 | 0.000 | 0.000 |
| **Lane 4** | 0.003 | 54.264 | 0.000 | 0.006 |

**Figure S8.** Raw data for western blots in Figures 5 and 6. (A) Chemiluminescence (left) and visible light (right) images of the USP22 and Cyclophilin B western blots shown in Figure 5A. Black rectangles indicate cropped sections shown in Figure 5A. Densitometry analyses for USP22 and Cyclophilin-B are indicated. The ratio of USP22/Cyclophilin-B is shown for each lane, as are the normalized ratios, which are presented relative to the siControl (Lane 1). **(B)** Chemiluminescence (left) and visible light (right) images of the USP22 and Cyclophilin B western blots shown in Figure 6A. Black rectangles indicate cropped sections shown in Figure 6A. Densitometry analyses for USP22 and Cyclophilin-B are indicated. The ratio of USP22/Cyclophilin-B is shown for each lane, as are the normalized ratios, which are presented relative to the Control (Lane 2).

## Supplementary Tables

**Table S1.** *USP22* silencing corresponds with global increases in H2Bub1 levels within prophase HCT116 cells.

| Condition | Number of cells analyzed | Median H2Bub1 signal (a.u.) | *p*-Value [A] |
|---|---|---|---|
| Untreated | 37 | 935 | 0.1553 |
| siControl | 39 | 700 | N/A |
| siUSP22-Pool | 41 | 1210 | 0.0001 |
| siUSP22-3 | 26 | 1431 | <0.0001 |

[A] Mann-Whitney tests compared to siControl (N/A not applicable). A p-value < 0.05 is considered significant.

**Table S2.** *USP22* silencing increases the frequency of chromatin compaction defects within mitotic HCT116 cells.

| Mitotic stage | Category | siControl | siUSP22-Pool |
|---|---|---|---|
| Prometaphase | Normal | 100% (*n* = 15) | 56% (*n* = 5) |
| | Compaction defect | 0% (*n* = 0) | 44% (*n* = 4) |
| Metaphase | Normal | 100% (*n* = 4) | 50% (*n* = 3) |
| | Compaction defect | 0% (*n* = 0) | 50% (*n* = 3) |

**Table S3.** *USP22* silencing induces significant increases in BUB1 recruitment to kinetochores within metaphase HCT116 cells.

| Condition | Number of cells analyzed | Median BUB1 signal (a.u.) | *p*-Value [A] |
|---|---|---|---|
| Vehicle control | 11 | 15.31 | N/A |
| Taxol | 10 | 46.56 | 0.0011 |
| Untreated | 46 | 14.76 | 0.0746 |
| siControl | 45 | 10.29 | N/A |
| siUSP22-Pool | 43 | 26.95 | <0.0001 |
| siUSP22-3 | 45 | 25.53 | <0.0001 |

[A] Mann-Whitney tests compared to vehicle control (Taxol) or siControl (Untreated, siUSP22-Pool, siUSP22-3). A p-value < 0.05 is considered significant (N/A not applicable).

**Table S4.** Mann-Whitney tests reveal significant increases in micronucleus formation following *USP22* silencing in HCT116 cells.

| Condition | Number of replicates [A] | Median number of micronuclei per 100 nuclei | Median fold change [B] | *p*-Value [C] |
|---|---|---|---|---|
| Untreated | 5 | 0.408 | 1.205 | 0.4286 |
| siControl | 6 | 0.339 | 1.000 | N/A |
| siUSP22-Pool | 6 | 0.789 | 2.328 | 0.0260 |
| siUSP22-2 | 6 | 0.638 | 1.882 | 0.0022 |
| siUSP22-3 | 6 | 1.349 | 3.980 | 0.0022 |

[A] A minimum of 100 nuclei/per replicate was analyzed. One replicate containing too few cells was excluded from the analysis in the Untreated condition (5 replicates remaining). [B] Median fold-change in micronucleus formation relative to siControl. [C] Mann-Whitney test compared to siControl (N/A not applicable). A p-value < 0.05 is considered significant.

**Table S5.** KS tests identify statistically significant changes in nuclear areas following *USP22* silencing.

| Cell line | Condition | Number of nuclei analyzed | Median (μm²) | *p*-Value [A] |
|---|---|---|---|---|
| HCT116 | Untreated | 1672 | 136 | 0.031 |
| | siControl | 1400 | 136 | N/A |
| | siUSP22-Pool | 1554 | 119.5 | <0.0001 |
| | siUSP22-2 | 1067 | 143 | <0.0001 |
| | siUSP22-3 | 619 | 148 | <0.0001 |
| hTERT | Untreated | 973 | 261 | 0.5689 |
| | siControl | 1171 | 257 | N/A |
| | siUSP22-Pool | 1362 | 269 | 0.0012 |
| | siUSP22-2 | 1174 | 255 | 0.0060 |
| | siUSP22-3 | 991 | 276 | <0.0001 |

[A]Two sample KS tests compared to siControl. A p-value < 0.01 is considered significant.

**Table S6.** *USP22* silencing induces numerical changes in chromosome complements.

| Cell line | Condition | Number of replicates | Spreads per replicate | Mean percentage of abnormal spreads | *p*-Value [A] |
|---|---|---|---|---|---|
| HCT116 | Untreated | 3 | 100 | 35 | 0.6520 |
| | siControl | 3 | 100 | 36 | N/A |
| | siUSP22-Pool | 3 | 100 | 46 | 0.0114 |
| | siUSP22-2 | 3 | 100 | 50 | 0.0050 |
| | siUSP22-3 | 3 | 100 | 57 | 0.0090 |
| hTERT | Untreated | 3 | 100 | 24 | 0.3703 |
| | siControl | 3 | 100 | 23 | N/A |
| | siUSP22-Pool | 3 | 100 | 40 | 0.0292 |
| | siUSP22-2 | 3 | 100 | 38 | 0.0143 |
| | siUSP22-3 | 3 | 100 | 40 | 0.0028 |

[A]Student's *t* tests compared to siControl. A p-value < 0.05 is considered significant..

**Table S7.** Mann-Whitney tests reveal significant changes in micronucleus formation in *USP22*-KO clones.

| Time point | Cell line | Number of replicates [A] | Median number of micronuclei per 100 nuclei | Median Fold Change [B] | *p*-Value [C] |
|---|---|---|---|---|---|
| Week 3 | Control | 6 | 0.960 | 1.000 | N/A |
| | *USP22*-KO-A | 5 | 1.521 | 1.584 | 0.3290 |
| | *USP22*-KO-B | 6 | 2.265 | 2.359 | 0.0087 |
| Week 5 | Control | 6 | 0.532 | 1.000 | N/A |
| | *USP22*-KO-A | 6 | 1.118 | 2.102 | 0.0260 |
| | *USP22*-KO-B | 6 | 1.304 | 2.446 | 0.0152 |
| Week 10 | Control | 6 | 0.496 | 1.000 | N/A |
| | *USP22*-KO-A | 6 | 0.796 | 1.607 | 0.2403 |
| | *USP22*-KO-B | 6 | 0.875 | 1.765 | 0.1320 |

[A]A minimum of 100 nuclei/per replicate was analyzed. One replicate containing too few cells was excluded from the analysis in the *USP22*-KO-A week 3 condition (5 replicates remaining). [B]Median fold-change in micronucleus formation

relative to Control cell line. [C]Mann-Whitney test p-value compared to Control (N/A not applicable). A p-value < 0.05 is considered significant.

**Table S8.** KS tests reveal significant changes in nuclear areas in *USP22*-KO clones.

| Time point | Cell line | Number [A] | Median (μm²) | *p*-Value [B] |
|---|---|---|---|---|
| Week 3 | Control | 300 | 169 | N/A |
| | *USP22*-KO-A | 300 | 166.5 | 0.5176 |
| | *USP22*-KO-B | 300 | 151 | <0.0001 |
| Week 5 | Control | 300 | 171 | N/A |
| | *USP22*-KO-A | 300 | 160 | 0.0209 |
| | *USP22*-KO-B | 300 | 174 | 0.8475 |
| Week 10 | Control | 300 | 124.5 | N/A |
| | *USP22*-KO-A | 300 | 141.5 | <0.0001 |
| | *USP22*-KO-B | 300 | 143 | <0.0001 |

[A]Number of nuclei analyzed. [B]Two sample KS tests compared to control (N/A not applicable). A p-value < 0.01 is considered significant.

**Table S9.** *USP22*-KO clones exhibit dynamic changes in chromosome numbers.

| Time point | Cell line | Number of spreads evaluated | Percentage of abnormal spreads |
|---|---|---|---|
| Week 3 | Control | 100 | 22% |
| | *USP22*-KO-A | 100 | 32% |
| | *USP22*-KO-B | 100 | 38% |
| Week 5 | Control | 100 | 29% |
| | *USP22*-KO-A | 100 | 21% |
| | *USP22*-KO-B | 100 | 29% |
| Week 10 | Control | 100 | 24% |
| | *USP22*-KO-A | 100 | 39% |
| | *USP22*-KO-B | 100 | 30% |

**Table S10.** Antibody sources and dilutions.

| Epitope | Supplier | Catalog Number | Species | Western Blot Dilution | Indirect Immunofluorescence Dilution |
|---|---|---|---|---|---|
| H2B | Abcam | ab1790 | Rabbit | 1:4,000 | NA |
| H2Bub1 | MilliporeSigma | 05-1312 | Mouse | 1:2,000 | 1:200 |
| USP22 | Sigma | HPA044980 | Rabbit | 1:12,000 | 1:200 |
| α-tubulin | Abcam | ab7291 | Mouse | 1:20,000 | NA |
| Cyclophilin-B | Abcam | ab16045 | Rabbit | 1:25,000 | NA |
| Anti-Rabbit HRP | Jackson ImmunoResearch | 111-035-144 | Goat | 1:15,000 | NA |
| Anti-Mouse HRP | Jackson ImmunoResearch | 115-035-146 | Goat | 1:10,000 | NA |
| PhosS10 | Abcam | ab32107 | Rabbit | NA | 1:1,000 |
| ACA | Center for Disease Control | IS2134 ANA#8 | Human | NA | 1:200 |
| BUB1 | Abcam | ab195268 | Rabbit | NA | 1:200 |
| Anti-Rabbit Cy3 | Jackson ImmunoResearch | 111-165-144 | Goat | NA | 1:200 |
| Anti-Mouse Alexa 488 | ThermoFisher Scientific | A-11029 | Goat | NA | 1:200 |
| Anti-Human Alexa 488 | ThermoFisher Scientific | A-11013 | Goat | NA | 1:200 |
